# Targeted Lipidomics and Inflammation Response to Six Weeks of Sprint Interval Training in Male Adolescents

**DOI:** 10.3390/ijerph20043329

**Published:** 2023-02-14

**Authors:** Aozhe Wang, Haifeng Zhang, Jianming Liu, Zhiyi Yan, Yaqi Sun, Wantang Su, Ji-Guo Yu, Jing Mi, Li Zhao

**Affiliations:** 1 Department of Exercise Physiology, Beijing Sport University, Beijing 100084, China; 2 Beijing Municipal Key Laboratory of Child Development and Nutriomics, Capital Institute of Pediatrics, Beijing 100020, China; 3 School of Competitive Sports, Beijing Sport University, Beijing 100084, China; 4 Department of Community Medicine and Rehabilitation, Faculty of Medicine, Umeå University, 901 87 Umeå, Sweden

**Keywords:** sprint interval training, inflammation, lipidomics, male adolescent

## Abstract

Lipids play an important role in coordinating and regulating metabolic and inflammatory processes. Sprint interval training (SIT) is widely used to improve sports performance and health outcomes, but the current understanding of SIT-induced lipid metabolism and the corresponding systemic inflammatory status modification remains controversial and limited, especially in male adolescents. To answer these questions, twelve untrained male adolescents were recruited and underwent 6 weeks of SIT. The pre- and post-training testing included analyses of peak oxygen consumption (VO_2_peak), biometric data (weight and body composition), serum biochemical parameters (fasting blood glucose, total cholesterol, high-density lipoprotein cholesterol, low-density lipoprotein cholesterol, triacylglycerol, testosterone, and cortisol), inflammatory markers, and targeted lipidomics. After the 6-week SIT, the serum C-reactive protein (CRP), interleukin (IL)-1β, IL-2, IL-4, IL-10, tumor necrosis factor (TNF)-α, and transforming growth factor (TGF)-β significantly decreased (*p* < 0.05), whereas IL-6 and IL-10/TNF-α significantly increased (*p* < 0.05). In addition, the targeted lipidomics revealed changes in 296 lipids, of which 33 changed significantly (*p* < 0.05, fold change > 1.2 or <1/1.2). The correlation analysis revealed that the changes in the inflammatory markers were closely correlated with the changes in some of the lipids, such as LPC, HexCer, and FFA. In conclusion, the 6-week SIT induced significant changes in the inflammatory markers and circulating lipid composition, offering health benefits to the population.

## 1. Introduction

In the past few decades, with the economy blooming all over the world, the population of overweight and obese adolescents has increased dramatically [1,2]. Regular physical exercise has been widely used as a non-pharmaceutical strategy to prevent the occurrence of obesity. Sprint interval training (SIT) is a mode of high-intensity interval training and has been proven to be a time-efficient way of improving cardiorespiratory fitness [3,4,5,6], aerobic performance [7,8,9,10], and metabolic function [11,12]. Obesity is a typical metabolic syndrome and is closely associated with chronic inflammation [13]. Although previous studies have reported positive effects of acute SIT on physical performance and metabolic health in adolescents [14,15,16], there is less evidence available regarding the overall health benefits of long-term SIT in adolescents, especially from the perspective of circulatory lipid metabolic adaptions and inflammatory responses.

Many studies have investigated the effects of acute SIT on the immune system in adults. Typically, SIT can result in transient metabolic disturbances (i.e., intracellular lactate accumulation), which can help to create a transient, fertile milieu of pro- and anti-inflammatory signaling cytokines, such as interleukins (IL)-6 and -10, and tumor necrosis factor (TNF)-α [17]. However, the effects of SIT on systemic inflammatory status remain controversial, as some studies reported no significant effect of 7/8 weeks of SIT on circulating inflammatory markers in young healthy people [18] and the elderly [19], whereas another study found that 2 weeks of SIT improved SIT-induced muscle damage and inflammatory status in adults [20]. Nevertheless, no study has been performed to investigate the effects of SIT on systemic inflammatory status in adolescents.

Inflammation and lipid signaling are the intertwined modulators of homeostasis and immunity. Interleukin 4 can stimulate lipogenesis and inhibit lipolysis [21]. Furthermore, IL-10 can reduce single molecule (PAzPC)-mediated lipid metabolic responses in cardiomyocytes, thereby mitigating inflammation and cell death [22]. In addition, IL-6 derived from skeletal muscle can encourage lipolysis in adipose and liver tissues [23]. It was reported that TNF-α can rapidly increase ceramide content by increasing de novo ceramide synthesis [24], and the inflammatory response to acute exercise is a possible mechanism in the increase in muscle ceramides [25]. On the other hand, different lipid species have different regulatory effects on the inflammatory response. Fatty acids (FAs) are involved in inflammatory signaling and play an important role in maintaining inflammatory homeostasis [26]. Despite these studies, however, the knowledge of the interaction between lipid and inflammatory signaling under the effects of chronic SIT is still limited, especially regarding adolescents.

Lipids are important components of the biomembrane, participate in numerous signaling pathways, including inflammatory pathways, and are a major fuel source for energy supply. Exercise regulates the rate of FA oxidation [27], as evidenced by acylcarnitine and FA accumulation immediately after exercise, accompanied by a transient accumulation of various complex lipids [28]. Furthermore, in trained male runners, long- and medium-chain FAs and FA oxidation products were significantly increased after exhaustive exercise [29]. Using lipidomic analysis [30], Wang et al. [31] found that TGs and apolipoprotein C3 were significantly decreased, whereas high-density lipoprotein cholesterol (HDL-C) was significantly increased after 8 weeks of aerobic exercise in patients with coronary heart disease. Another study, on women with obesity [32], found that 12 weeks of combined aerobic and resistance exercise training significantly altered the skeletal muscle lipid profile. Therefore, the lipidomic technique is a powerful tool in helping us to understand the impact of exercise on lipid trajectories. However, most of the studies on this topic focused on patients or elite athletes, and very few studies paid attention to adolescents with respect to lipidomic characteristics under the effects of high-intensity exercise.

Therefore, the purpose of this study is to investigate the effects of 6 weeks of SIT on circulatory lipid profiles and inflammatory reactions in adolescents, as well as their interactions.

## 2. Materials and Methods

### 2.1. Participants

Fifteen male adolescents (aged 12–14 years) volunteered to participate in this study. All the participants were from the same school, with similar ages, BMIs, living environments, diets, sleep and waking rhythms, class schedules, and recreational activities. The inclusion criteria were (1) no cardiovascular, pulmonary, or metabolic disease; (2) no inflammation-related disease; and (3) no regular physical training for the past year. The anthropometry, body mass index (BMI), and body composition (Inbody720, Korea) were measured and recorded.

Twelve of the participants (age 12.9 ± 0.8 years; height 163.29 ± 8.65 cm) completed a 6-week SIT program and all the tests both before and after the training. Before the study, the experimental procedure and potential risks were fully explained to the participants, and written informed consent was obtained from all of them. The experimental protocol was approved by the Ethics Committee of Beijing Sport University (2020139H) and was carried out in accordance with the Declaration of Helsinki.

### 2.2. Training Protocol

The SIT training consisted of repeated Wingate tests on an ergometer (Monark 894E, Monark, Sweden), 3 days per week for 6 consecutive weeks. Training sessions started with a short period (10–15 min) of warm-up on the ergometer at 60 rpm and 25 W. Next, the participants performed 30 s sprint cycling (maximal speed) at a resistance of 7.5% body weight (kg). In the first and second weeks of the SIT training, the sprint cycling was repeated 4 times per training session, and from week 3 to week 6, sprint cycling was repeated 6 times per training session, with 4 min rest intervals between repetitions. During the rest period, the participants pedaled at 60 rpm and 25 W.

### 2.3. Blood-Sample Collection and Analysis

The participants were asked to refrain from alcohol, caffeine, dietary supplements, and strenuous exercise for at least 48 h before blood sampling. Blood samples were obtained after an overnight fast from all the participants before and 48 h after the last session of SIT training at approximately the same time of the day (8:00 a.m.). The blood samples were obtained through venipuncture and collected in procoagulant-containing tubes (serum tubes). Samples in serum tubes were allowed to clot for 30 min at room temperature. Samples were centrifuged at 4 °C and 3000 rpm for 10 min. Serum was then extracted, frozen in liquid nitrogen for 3–5 s, and stored at −80 °C for further analysis.

### 2.4. Peak Oxygen Consumption (VO_2_peak)

The participants undertook a standard warm-up (5 min) on an ergometer (894E, Monark, Vansbro, Sweden), followed by a 3 min rest. The VO_2_peak was evaluated using a metabolic gas analysis system (Cortex MetaMax 3B, Cortex, Isernhagen, Germany), and heart rates (HR) of the participants were monitored using a chest-worn monitor (Polar H10, Polar, Kempele, Finland). During the VO_2_peak test, the participants first performed 1 min of cycling at a speed of 60 rpm without loading, followed by 2 min of cycling at a loading of 25 W; thereafter, the loading was increased by 25 W every 2 min until exhaustion. The VO_2_peak was reached when any two of the following three criteria were met: (1) the respiratory quotient was higher than 1.10; (2) the HR reached 90% of the predicted personal maximum; and (3) the participant reached exhaustion.

### 2.5. Serum Biochemical Characteristics

Serum concentrations of TG, total cholesterol (TCHO), low-density lipoprotein cholesterol (LDL-C), and HDL-C were measured using a biochemical analyzer, Beckman Coulter AU2700 (AU2700, Beckman Coulter, Brea, CA, USA). Fasting blood glucose (FBG) concentration was measured with a glucose dehydrogenase assay (ACCU-CHEK Active Blood Glucose Meter, Roche Diabetes, Mannheim, Germany), and serum testosterone (Testo) and cortisol were measured via chemiluminescence immunoassay (ACCESS 2, Beckman Coulter, Brea, CA, USA).

### 2.6. Inflammatory Markers

Serum concentrations of IL-1β, IL-2, IL-4, IL-6, and IL-10 and transforming growth factor (TGF)-β and TNF-α were assayed with enzyme-linked immunosorbent assay (ELISA) kits (Shanghai Jianglai Industrial Limited by Share Ltd., Shanghai, China). The serum concentration of C-reactive protein (CRP) was measured using a biochemical analyzer, Beckman Coulter AU2700 (AU2700, Beckman Coulter, Brea, CA, USA).

### 2.7. Targeted Lipidomic Analysis

For targeted lipidomics, a semi-quantitative approach was used. Lipids were extracted from 100 µL serum samples using simple protein precipitation in pre-chilled isopropanol (IPA) at 4 °C. The samples were mixed with lipid internal standard, after which 500 µL of pre-chilled IPA was added, vortexed for 1 min, placed at −20 °C for 10 min, and vortexed again for 10 min. After 2 h at 4 °C, the mixture was centrifuged at 10,300× *g* for 10 min at 4 °C, and the supernatant was removed. All analyses were performed in electron spray ionization (ESI) mode using a Waters iclass-Xevo TQ-S ultra-high-performance liquid chromatography–tandem mass spectrometry system (Waters, Milford, MA, USA). The dwell time for each lipid species was 3 ms. The source nitrogen temperature was 120 °C, and the flow rate was 150 L/h. The desolvation gas temperature was 500 °C, and the flow rate was 1000 L/h. The capillary voltage was 2.8 kV in the positive mode and 1.9 kV in the negative mode. The autosampler operated at 4 °C and the column chamber at 45 °C during the analysis.

Lipid species were separated using a Waters Acquity UPLC BEH Amide column (1.7 μm, 2.1 × 100 mm). Solvent A was 95% acetonitrile containing 10 mM ammonium acetate, and solvent B was 50% acetonitrile containing 10 mM ammonium acetate. The mobile-phase gradient was 0.1–20% B for 2 min, followed by 20–80% B for 3 min and 3 min re-equilibration, with a flow rate of 0.6 mL/min. Mass spectrometry multiple-reaction monitoring (MRM) was established for the identification and quantitative analysis of various lipids. Individual lipids were quantitated relative to their respective internal standards, including d7-phosphatidylcholine (15:0/18:1), d7-phosphatidylethanolamine (15:0/18:1), d7-phosphatidylglycerol (15:0/18:1), d7-phosphatidylinositol (15:0/18:1), d7-lysophosphatidylcholines (15:0/18:1), d7-lysophosphatidylethanolamine (15:0/18:1), d7-diacylglycerols (15:0/18:1), d7- triacylglycerols (15:0/18:1), d9-sphingomyelin (18:1), d7-cholesteryl esters (18:1), and d7-monoacylglycerols (18:1) obtained from Avanti Polar Lipids, and d7-phosphatidic acids (15:0/18:1) from Sigma-Aldrich.

### 2.8. Statistical Analyses

TargeLynx in MassLynx v4.1 (Waters, Milford, MA, USA) was used to process the lipidomic raw data. Each peak was automatically integrated but confirmed and adjusted manually if needed. The retention time of each lipid class internal standard was used to confirm peak integration for the lipids belonging to the same class. Lipids with missing values greater than 50% were removed, and the multiple-interpolation method was used to fill in the missing values for the remaining lipids. All lipids were log-transformed to approximate a normal distribution. Lipidomic data were analyzed using a paired-sample *t*-test, with a significance level set at *p* ≤ 0.05 and a 95% confidence interval (CI). Principal component analysis (PCA) was used to observe the degree of sample dispersion. The correlations between the significantly changed lipids and inflammatory markers were evaluated using the Spearman correlation analysis.

## 3. Results

### 3.1. Participant Characteristics in Response to the 6 Weeks of SIT

The biometric data and serum biochemical measurements are presented in Table 1. The weight, BMI, muscle mass, and VO_2_peak of the participants increased significantly (*p* < 0.01) after 6 weeks of SIT. The concentrations of TCHO, HDL-C, LDL-C, TG, and FBG were at normal serum levels and did not present significant changes. The 6 weeks of SIT did not cause a notable change in Testo or cortisol but resulted in a significant increase in T/C (*p* < 0.05).

### 3.2. Inflammatory Markers

The results of the inflammatory markers are presented in Table 2. The 6 weeks of SIT resulted in significant changes in all the circulating inflammatory markers. The concentrations of CRP (*p* < 0.01), IL-1β (*p* < 0.01), IL-2 (*p* < 0.01), IL-4 (*p* < 0.01), IL-10 (*p* < 0.001), TNF-α (*p* < 0.001), and TGF-β (*p* < 0.001) decreased significantly. By contrast, the concentrations of IL-6 (*p* < 0.01) increased significantly. The IL-10/TNF-α values also increased significantly after 6 weeks of training (*p* < 0.05).

### 3.3. Widespread Changes in the Lipidomics Pre- and Post-SIT

**Figure 1:**
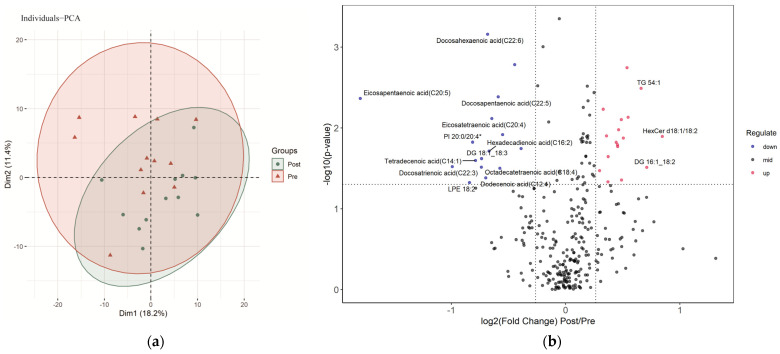
Characterization of serum lipids pre- and post-sprint interval training: (**a**) PCA (principal component analysis) of serum lipids pre- and post-sprint interval training; (**b**) volcano plot demonstrating the changes in serum lipids induced by sprint interval training. Lipids with labeling fold change > 1.5 or <1/1.5 and *p* < 0.05.

The PCA plot did not show a distinct separation in lipidomics between the samples before and after the SIT (Figure 1a). The 6 weeks of SIT resulted in significant differences in serum lipids (33 of 296, 11%), 16 of which decreased significantly (*p* < 0.05, fold change < 1/1.2), and 17 increased significantly (*p* < 0.05, fold change > 1.2), as shown in Figure 1b. A detailed list of the differential lipids, including their names, subclasses, categories, and fold changes, is reported in Supplemental Table S1.

Out of the 27 quantified free fatty acids (FFAs), 11 significantly differed in pre-and post-training. More serum long-chain FFAs than medium-chain FFAs significantly changed after the 6 weeks of SIT. The serum dodecenoic acid (C12:1), myristic acid (C14:0), tetradecenoic acid (C14:1), hexadecadienoic acid (C16:2), octadecatrienoic acid (C18:3), octadecatetraenoic acid (C18:4), eicosatetraenoic acid (C20:4), eicosapentaenoic acid (C20:5), docosatrienoic acid (C22:3), docosapentaenoic acid (C22:5), and docosahexaenoic acid (C22:6) decreased significantly after the 6 weeks of training. Eicosapentaenoic acid (C20:5) had the largest amplitude of change.

The SIT caused widespread changes in the neutral lipids. Serum CE 18:2 was significantly reduced after the training, while serum MG 22:5 increased significantly. We quantified 39 DGs, 6 of which were significantly different after the training. Serum DG 14:0_16:0, DG 16:0_16:0, DG 16:0_18:0, DG 18:0_18:0, and DG 16:1_18:2 were higher after training, while serum DG 18:1_18:3 was lower. Out of the 20 quantified TGs, 4 significantly changed in response to the 6 weeks of SIT. The serum TG 50:0, TG 52:2, TG 54:1, and TG 54:2 increased significantly.

The SIT resulted in significant increases in serum Cer d18:1/16:0, Cer d18:1/18:1, HexCer d18:1/14:0, HexCer d18:1/18:2, and HexCer d18:1/16:1.

Furthermore, the SIT also caused significant changes in the serum concentrations of specific PC, phosphatidylinositol (PI), lysophosphatidylcholine (LPC), and lysophosphatidylethanolamine (LPE). Serum PI 18:0/18:0 and PI 16:0/18:0* increased significantly after the training, while serum PI 20:0/20:4*, LPC 20:0, and LPE 18:2 decreased significantly.

### 3.4. Lipidomic Changes Associated with Changes in Circulating Inflammatory Markers

**Figure 2:**
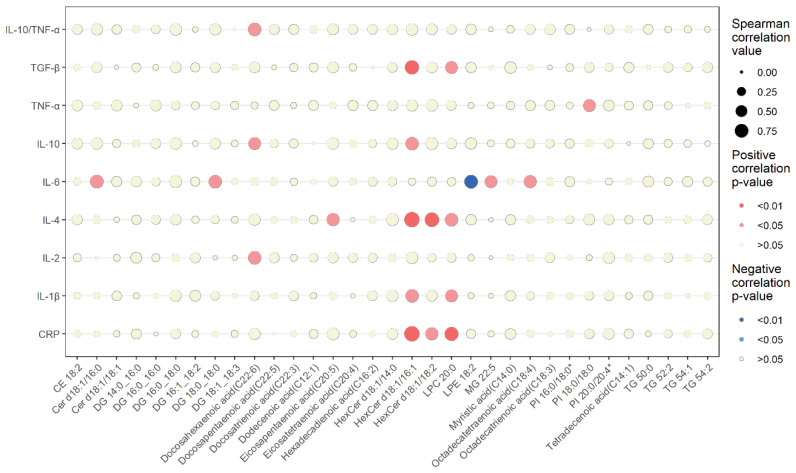
Spearman correlations between changes in differential serum lipids and inflammatory markers. The dot size represents the correlation value. The shade of the dot color represents significance (blue, negative correlation; red, positive correlation).

The relationships between the changes in the differential serum lipids and circulating inflammatory markers are presented in Figure 2. A detailed list of the Spearman correlation analysis is reported in Supplemental Table S2. The decrease in CRP was strongly and positively correlated with the decrease in LPC 20:0 and the increase in HexCer d18:1/16:1, and moderately correlated with the increase in HexCer d18:1/18:2. The decrease in IL-1β was positively correlated with the decrease in LPC 20:0 and the increase in HexCer d18:1/16:1. The decrease in TNF-α was positively correlated with the increase in PI 18:0/18:0.

The decrease in IL-4 was strongly and positively correlated with the increases in both HexCer d18:1/16:1 and HexCer d18:1/18:2 and moderately positively correlated with the decreases in LPC 20:0 and eicosapentaenoic acid (C20:5). The decreases in IL-2, IL-10, and IL10/TNF-α were positively correlated with the decrease in docosahexaenoic acid (C22:6). In addition, the increase in IL-10 was positively correlated with the increase in HexCer d18:1/16:1. The decrease in TGF-β was positively correlated with the decrease in LPC 20:0 and the increase in HexCer d18:1/16:1.

A higher number of changes in lipids showed an association with IL-6 changes compared with the other inflammatory markers. The increase in IL-6 was positively correlated with increases in Cer d18:1/16:0, DG 18:0_18:0, and MG 22:5 and the decrease in octadecatetraenoic acid (C18:4). The increase in IL-6 was also negatively correlated with the decrease in LPE 18:2.

## 4. Discussion

The present study documented, for the first time, the effects of 6 weeks of SIT on the circulating inflammatory markers and targeted lipidomics in male adolescents. Significant increases were observed in body weight, BMI, and muscle mass after the SIT, while the serum levels of TCHO, HDL-C, TG, and FBG were not significantly altered, indicating that the increases in body weight were most likely due to the increase in muscle mass or lean body mass caused by the training. In agreement with previous studies [10,33,34], the SIT improved the VO_2_peak with a significant increase in muscle mass in adolescents. There was no significant change in Testo or cortisol, but a significant increase in T/C was observed, potentially reflecting enhancements in metabolism due to the training. The SIT also resulted in significant increases in IL-6 and the IL-10/TNF-α ratio and decreases in the other pro-inflammatory and anti-inflammatory cytokines. These results indicate that the SIT is efficient in improving serum lipid metabolism and inflammatory reactions, thus improving the health status of male adolescents.

### 4.1. Changes in Circulating Inflammatory Markers Pre- and Post-SIT

Evidence suggests that long-term exercise can promote an anti-inflammatory status, which appears to be the key factor in reducing chronic diseases [35,36]. In this study, we demonstrated, for the first time, that 6 weeks of SIT significantly decreased the circulating inflammatory markers CRP, IL-1β, IL-2, IL-4, IL-10, TNF-α, and TGF-β, and increased IL-6 in adolescents. We also found there was an increase in the IL-10/TNF-α ratio post-SIT, which was positively associated with anti-inflammatory profiles. The activation of the immune system results in the release of cytokines that are classified into pro- (i.e., IL-1β, TNF-α, and IL-8) or anti-inflammatory (i.e., IL-2, IL-4, IL-10, and TGF-β) factors [37]. Interleukin 6 has not only proinflammatory but also anti-inflammatory activity [38]. Inflammatory-marker interactions maintain inflammatory homeostasis. The CRP is the prototypical biomarker for inflammation that can promote the secretion of IL-1β and IL-6 [39]. Furthermore, TNF-α also stimulates the production of IL-6, and IL-6 inhibits the production of TNF-α. In addition, TNF-α induces insulin resistance and lipolysis without affecting fat oxidation [40], whereas IL-6 has the opposite effect. The increased circulating IL-6 levels from contracting skeletal muscle may enhance insulin sensitivity [41], lipolysis, and fat oxidation [36], therefore reducing the risk of metabolic disorders [42]. Interleukins 4 and 10 are released into circulation in order to neutralize the proinflammatory cytokine cascade, inhibiting the production of proinflammatory cytokines. Interleukins 2 and 4 synergistically promote the production of Tregs and increase the production of IL-10, leading to an even more robust ability to suppress overactive immune responses [43]. The TGF-β can induce both regulatory/inhibitory and proinflammatory T cells, depending on proinflammatory cytokines [44]. Many research reports show that the protective effect of regular exercise may be due to reduced inflammatory immune profiles, characterized by reduced proinflammatory levels and raised IL-6 and anti-inflammatory levels in aged or at-risk populations [42,45,46]. Similarly, we found that the SIT stimulated increases in the IL-6 concentration and the IL-10/TNF-α ratio. However, the other inflammatory cytokines, either proinflammatory or anti-inflammatory factors, were initially found to have decreased. It was demonstrated that muscle-derived IL-6 increases with exercise in the absence of increased TNF-α and IL-1β in the latter state [47]. Therefore, the current study’s results suggest that increased IL-6 exerts an anti-inflammatory effect. Together with the increase in the IL-10/TNF-α ratio, this results in a positive training adaptation of the immune system. Accordingly, this may explain the reason why the inflammatory immune profiles of the adolescents were somewhat improved compared with before the SIT.

### 4.2. Changes in Lipidomics Pre- and Post-SIT

The greater effort evoked by SIT stimulates a lipidomic disturbance, which may induce considerable physiological changes in the immune system. We hypothesized that SIT would increase lipid utilization and alter lipid intermediates. Therefore, a targeted lipidomic analysis of the serum samples was used to reveal detailed insights into the effects of the SIT on lipid metabolism. The 6 weeks of SIT resulted in decreased serum FFA levels, especially in the species of dodecenoic acid (C12:1), myristic acid (C14:0), tetradecenoic acid (C14:1), hexadecadienoic acid (C16:2), octadecatrienoic acid (C18:3), octadecatetraenoic acid (C18:4), eicosatetraenoic acid (C20:4), eicosapentaenoic acid (C20:5), docosatrienoic acid (C22:3), docosapentaenoic acid (C22:5), and docosahexaenoic acid (C22:6). Serum myristic acid (C14:0) is considered a biomarker for predicting TCHO [48,49] and a new candidate marker for severe inflammation [50]. We found that the 6 weeks of SIT did not cause significant changes in TCHO but resulted in a significant decrease in serum myristic acid (C14:0) levels, which suggested that SIT may have a potential effect on low levels of TCHO. Free fatty acids are essential energy substrates during endurance exercise. The demand for FAs as energy sources during exercise is met by the hydrolysis of IMTG and the systemic supply of lipids originating from the adipose tissue, the small intestine, and the liver [51]. In addition, the utilization of blood FFAs in working muscles is also important for aerobic ATP synthesis during prolonged exercise of moderate intensity [52]. Whilst high intra- and/or extracellular FA concentrations exceed physiological levels, excess FAs exert lipotoxicity. High FAs favor a proinflammatory status and contribute to the development of insulin resistance [53,54]. Thus, we believe that the decrease in serum FFA levels may have supported the efficacy of the SIT in preserving insulin sensitivity in adolescents.

Usually, in order to prevent lipotoxicity, excessive FFAs are converted into neutral lipids, such as TGs and sterol esters, for storage. During TG lipolytic processes, the FFAs are liberated, and DGs and MGs are generated in sequence; alternatively, these FAs can be re-esterified back into TGs for storage [55].

In our study, the 6 weeks of SIT induced a decrease in serum DG 18:1_18:3 and increases in DG 14:0_16:0, DG 16:0_16:0, DG 16:0_18:0, DG 18:0_18:0, DG 16:1_18:2, TG 50:0, TG 52:2, TG 54:1, and TG 54:2. It has been reported that during exercise, TG oxidation is greater, and during recovery, plasma FFAs decrease more rapidly in trained subjects than in untrained subjects [56]. Given that TGs are efficient and inert forms of FA for storage and transport, the aforementioned increase in glycerolipids may indicate that 6 weeks of SIT facilitates FFA re-esterification to maintain decreased circulating FFA levels with altered lipid intermediates in trained adolescents.

Ceramide, a sphingosine derivative, constitutes the hydrophobic backbone of all complex sphingolipids (e.g., SM, cerebrosides, and gangliosides). The hydrolysis of SM by sphingomyelinase (SMase) is the main process responsible for ceramide generation in the plasma membrane [57]. Furthermore, SM and ceramide are fundamental structural components of cell membranes, and they are involved in the regulation of signaling pathways in cell growth, differentiation, and apoptosis, even regulating inflammatory response [58]. In these cellular stress conditions or in response to a wide variety of stimuli, SMase pathways are activated, leading to rapid ceramide formation in the plasma membrane [59]. Subsequently, ceramide’s association with SM changes the cell membrane lateral pressure profile, resulting in decreases in their lateral diffusion, thereby stabilizing these signaling molecule interactions. This swift ceramide generation was found on the surfaces of T and B cells following the activation of TNF receptor family members [60]. In our study, the SIT increased the contents of Cer d18:1/16:0, Cer d18:1/18:1, HexCer d18:1/14:0, HexCer d18:1/18:2, and HexCer d18:1/16:1 in the serum. The structural features of the ceramide species might control its specificity toward a specific cellular process. It was reported that C18-ceramides are linked to increased mitophagy and cell death [58], and C16-ceramides are associated with glucose intolerance and apoptosis [61]. We believe that increases in the C16:0 and C18:1 ceramides are associated with SIT-induced inflammatory response to facilitating the ligand-induced clustering of these receptors.

Furthermore, we found that the SIT significantly decreased PI 20:0/20:4*, LPC 20:0, and LPE 18:2 and significantly increased PI 18:0/18:0 and PI 16:0/18:0*. Lysophosphatidylcholine is a major lipid component of oxidized low-density lipoprotein and plays different roles in the regulation of inflammation by binding to G-protein-coupled receptors and Toll-like receptors (TLRs). For example, LPC can induce insulin resistance; on the other hand, it can decrease blood glucose [62]. Lysophosphatidylethanolamine is a minor component of the cell membrane [63]. Lysophosphatidylcholine and LPE are both involved in the regulation of the inflammatory response system [64]. Lysophosphatidylcholine can trigger TLR2- and TLR4-mediated signaling pathways. Lysophosphatidylethanolamine was recently reported to be involved in anti-inflammatory effects on macrophage polarization induced by lipopolysaccharide [65]. This study found that the 6 weeks of SIT resulted in a significant reduction in adolescent serum LPC 20:0 and LPE18:2, suggesting that SIT may affect adolescent metabolic health and inflammatory status by modulating LPC and LPE.

Typically, PI is present in low amounts in cell membranes relative to other phospholipids. Phosphatidylinositol can be phosphorylated at several of its hydroxy sites to form PI subclasses. Although these phosphorylated PI species play several key roles in various facets of maintaining essential cellular functions [66], PI in serum is relatively sparsely reported. In the current study, the increases in PI 18:0/18:0 and PI 16:0/18:0* and the decrease in PI 20:0/20:4* may have also been associated with SIT-induced changes in inflammatory status. Further research is important to understand the role of these minor species in regulating characterization.

Finally, we observed a decrease in serum CE 18:2, which may have resulted from the SIT facilitating CE transport and delivery by affecting cholesteryl ester transfer protein [67]. Therefore, in our study, the numerous lipid intermediates described above were significantly different after the 6 weeks of SIT, but the total serum TCHO and TG concentrations were in the normal serum range. We took this finding to suggest that these altered lipid intermediates may be associated with SIT-induced changes in immune regulation.

### 4.3. Lipidomic Changes Associated with Changes in Circulating Inflammatory Markers

Given that lipid metabolism and inflammatory and innate immune processes are coordinated and regulated by exercise, we further explored whether the changes in serum lipidomics were related to changes in circulating inflammatory markers. We focused on the impact of the changes in differential lipid intermediates on the changes in inflammatory markers induced by the SIT.

Docosahexaenoic acid (C22:6), eicosapentaenoic acid (C20:5), and octadecatetraenoic acid (C18:4) are both *n*-3 polyunsaturated fatty acids (PUFAs). Octadecatetraenoic acid (C18:4) may be used as a precursor to the increase in the eicosapentaenoic acid (C20:5) content [68]. Although *n*-3 PUFAs can reduce inflammation; regulate the nervous system, blood pressure, and glucose tolerance; and help lower the risk of heart disease, cancer, and arthritis [69,70], it was found that the intake of exogenous long-chain *n*-3 PUFAs did not demonstrate marked effects on plasma inflammatory markers in healthy participants [71]. Recent studies have shown that *n*-3 PUFAs can affect not only exercise and skeletal-muscle metabolism but also the functional response to exercise training for a period of time [72]. Our study found that exercise-induced decreases in endogenous *n*-3 PUFAs were strongly associated with changes in inflammatory status. Docosahexaenoic acid (C22:6) and eicosapentaenoic acid (C20:5) showed consistent changes with the anti-inflammatory cytokines IL-2, IL-4, and IL-10, and the decrease in octadecatetraenoic acid (C18:4) was positively associated with the increase in IL-6. In addition, the decrease in docosahexaenoic acid (C22:6) was positively associated with the increase in IL-10/TNF-α. Combined with our findings of increased IL-6 and decreases in other anti- and proinflammatory cytokines, it is suggested that decreased *n*-3 PUFAs induced by SIT may help to maintain anti-inflammatory status in adolescents.

The MGs, DGs, and ceramides are the products of the hydrolysis of TGs or appear as lipid intermediate during the de novo synthesis of TGs. We found that increases in Cer d18:1/16:0, DG 18:0_18:0, and MG 22:5 were positively associated with the increase in IL-6. This indicated that the SIT-induced changes in specific MGs, DGs, and ceramides interacted with the increased IL-6. This was in accordance with previous findings showing that IL-6 plays a significant role in lipid metabolism by preventing obesity development [73]. We also found that the increase in HexCer d18:1/18:2 was positively associated with decreases in CRP and IL-4. At present, there is no direct evidence for the role of this specific HexCer d18:1/18:2 in the proinflammatory or anti-inflammatory response. Further experimental studies are required to determine the underlying mechanisms responsible for the association of HexCer d18:1/18:2 with the inflammatory response. Notably, we found that the increase in HexCer d18:1/16:1 showed a positive correlation with decreases in CRP, proinflammatory cytokines (IL-1β), and anti-inflammatory cytokines (IL-4, IL-10, and TGF-β). Therefore, we inferred that HexCer d18:1/16:1 is the key lipid linking lipid metabolism and inflammatory response, but its complex function and the specific mechanisms through which it operates remain to be explored in depth.

Although several studies have confirmed the role of lysophospholipids in the inflammatory response, their specific effects have not been conclusively established. Recent studies found that different subtypes of LPCs play opposite roles in the regulation of inflammation. Polyunsaturated acyl LPCs reduce inflammatory-cell activation and encourage the release of anti-inflammatory cytokines [74]. By contrast, saturated LPCs induce the release of proinflammatory cytokines [62]. It was reported that the administration of LPC and LPE to lipopolysaccharide (LPS)-stimulated SIM-A9 microglia cells significantly reduced the expression of IL-6 [75]. Our study found a negative correlation between the SIT-induced decrease in LPE 18:2 and the increase in IL-6, demonstrating the link between LPE and IL-6. Moreover, we found an association between LPC 20:0 and multiple inflammatory cytokines, with a positive correlation between the decrease in LPC 20:0 and decreases in CRP, IL-1β, IL-4, and TGF-β. We hypothesize that LPC 20:0 may be another important lipid linking lipid metabolism to the inflammatory response and maintaining the immune system in a steady state. In addition, we found that the SIT-induced increase in serum PI 18:0/18:0 was positively associated with changes in TNF-α, but the exact mechanism involved is unclear.

Altogether, the SIT altered *n*-3 PUFAs, MG, phospholipid, DG, and ceramide subtypes that were associated with changes in anti-inflammatory cytokines (IL-2, IL-4, IL-6, IL-10, and TGF-β) and proinflammatory markers (CRP, IL-1β, and TNF-α). Given that the SIT-induced activation of IL-6 may inhibit ongoing TNF-α transcription, blocking TNF-α leads to a subsequent decrease in the systemic levels of inflammatory cytokines. It is most likely that the anti-inflammatory environment resulting from SIT benefits adolescents with increased physical fitness.

A limitation of our study is that adolescents undergo critical periods of growth and development, which may compound the interpretation of results based only on blood samples, rather than on fat and skeletal tissue. However, given that cellular stress and the resultant metabolic adaptations depend largely on exercise intensity, our findings obtained from the blood samples reflected not only the status of the serum itself but also the status of other tissues. Of course, the small number of participants, the relatively short observation period, the lack of assessment of long-term changes, and the lack of assessment of serum insulin levels also need to be considered.

## 5. Conclusions

In conclusion, our study demonstrated that the 6 weeks of SIT did not alter the serum TCHO, HDL-C, LDL-C, or TG concentrations but resulted in significant increases in IL-6 and the IL-10/TNF-α ratio and led to decreases in all the other pro- and anti-inflammatory cytokines, which were closely correlated with the alterations in circulating lipid composition in adolescents. Furthermore, we found that HexCer d18:1/16:1 and LPC 20:0 were the key lipid intermediates that were closely correlated with the changes in the lipid profiles and inflammatory responses. This study provides additional evidence for the potential for SIT to elicit physiological adaptations, improving physical fitness in terms of modulating lipid metabolism and inflammation response in male adolescents. We propose that the SIT stimulated lipid utilization and altered the lipid intermediates associated with inflammation cytokines, increasing the anti-inflammatory status of adolescents. Further studies are required to determine whether changes in serum-specific lipid intermediates are essential in regulating inflammatory reactions and increasing the efficiency with which the occurrence of obesity and various metabolic diseases is prevented in adolescents.

## Figures and Tables

**Table 1:** Anthropometric, exercise, and biochemical characteristics pre- and post-sprint interval training.

	Pre	Post	*p*
Weight (kg)	55.36 ± 11.31	58.66 ± 11.37 **	0.000
BMI (kg/m^2^)	20.61 ± 2.99	21.61 ± 2.92 **	0.001
Muscle mass (kg)	24.98 ± 5.31	26.13 ± 5.33 **	0.001
VO_2_peak (L/min)	2.36 ± 0.35	2.81 ± 0.42 **	0.000
VO_2_peak (mL/min/kg)	43.25 ± 4.09	48.58 ± 5.02 **	0.000
TCHO (mol/L)	3.73 ± 0.68	3.93 ± 0.92	0.182
HDL-C (mol/L)	1.50 ± 0.26	1.59 ± 0.35	0.345
LDL-C (mol/L)	1.88 ± 0.48	2.11 ± 0.57	0.297
TG (mol/L)	0.79 ± 0.19	0.92 ± 0.32	0.231
FBG (mmol/L)	4.83 ± 0.26	4.94 ± 0.46	0.117
Testo (nmol/L)	10.16 ± 5.05	10.56 ± 4.87	0.583
Cortisol (nmol/L)	379.95 ± 85.76	329.73 ± 135.81	0.308
T/C	0.03 ± 0.02	0.04 ± 0.02 *	0.041

Data are means ± SD. BMI, body mass index; VO_2_peak, peak oxygen consumption; TCHO, total cholesterol; HDL-C, high-density lipoprotein cholesterol; LDL-C, low-density lipoprotein cholesterol; TG, triacylglycerol; FBG, fasting blood glucose; Testo, testosterone. Significantly different from pre-training: * *p* < 0.05; ** *p* < 0.01.

**Table 2:** Inflammatory markers pre- and post-sprint interval training.

	Pre	Post	*p*
CRP (mg/L)	1.85 ± 0.78	0.83 ± 0.27 **	0.001
IL-1β (pg/mL)	6.55 ± 4.22	1.95 ± 1.53 **	0.002
IL-2 (ng/mL)	0.34 ± 0.07	0.22 ± 0.04 **	0.002
IL-4 (pg/mL)	5.77 ± 3.06	2.48 ± 0.83 **	0.005
IL-6 (pg/mL)	1.06 ± 0.91	3.31 ± 1.03 **	0.003
IL-10 (pg/mL)	118.29 ± 44.32	45.99 ± 23.38 **	0.001
TNF-α (pg/mL)	74.58 ± 15.95	17.96 ± 2.27 **	0.000
TGF-β (pg/mL)	625.15 ± 365.04	122.28 ± 58.77 **	0.001
IL-10/TNF-α	1.64 ± 0.62	2.62 ± 1.40 *	0.048

Data are means ± SD. CRP, C-reactive protein; IL-1β, interleukin 1β; IL-2, interleukin 2; IL-4, interleukin 4; IL-6, interleukin 6; IL-10, interleukin 10; TNF-α, tumor necrosis factor-α; TGF-β, transforming growth factor-β. Significantly different from pre-training: * *p* < 0.05; ** *p* < 0.01.

## Data Availability

The datasets used and/or analyzed during the current study are available from the corresponding author upon reasonable request.

## Supplementary Materials

The following supporting information can be downloaded at: https://www.mdpi.com/article/10.3390/ijerph20043329/s1. Table S1: Fold change of differential serum lipids pre- and post-sprint interval training. Table S2: Spearman correlations between changes in differential serum lipids and inflammatory markers.
