# Supplementary material for: Targeted Lipidomics and Inflammation Response to Six Weeks of Sprint Interval Training in Male Adolescents
Source: Int J Environ Res Public Health. 2023 Feb 14;20(4):3329. doi: 10.3390/ijerph20043329 (PMC9963480; doi:10.3390/ijerph20043329)
Supplement: Supplementary file 1 [file ijerph-20-03329-s001.zip › Supplementary Table S1.pdf]

Table S1. Fold-change of differential serum lipids pre- and post-sprint interval training

Lipid Categories	Sub Class	Lipid Name	Fold Change	p-Value
Fatty Acyls (FA)	Saturated Fatty Acids (SFA)	Myristic acid	0.68	0.012
	Unsaturated Fatty Acids (UFA)	Eicosapentaenoic acid	0.29	0.004
		Docosatrienoic acid	0.50	0.030
		Tetradecenoic acid	0.58	0.025
		Octadecatetraenoic acid	0.60	0.031
		Dodecenoic acid	0.62	0.042
		Docosahexaenoic acid	0.62	0.001
		Hexadecadienoic acid	0.63	0.019
		Eicosatetraenoic acid	0.64	0.008
		Docosapentaenoic acid	0.66	0.004
		Octadecatrienoic acid	0.67	0.032
Glycerolipids (GL)	Monoacylglycerols (MG)	MG 22:5	1.29	0.047
	Diacylglycerols (DG)	DG 18:1_18:3	0.60	0.024
		DG 16:0_18:0	1.29	0.023
		DG 16:0_16:0	1.37	0.016
		DG 14:0_16:0	1.40	0.008
		DG 18:0_18:0	1.42	0.013
		DG 16:1_18:2	1.64	0.031
	Triacylglycerol (TG)	TG 52:2	1.23	0.034
		TG 54:2	1.45	0.002
		TG 50:0	1.46	0.007
		TG 54:1	1.58	0.003
Glycerophospholipids (GP)	Phosphatidylinositol (PI)	PI 20:0/20:4*	0.57	0.015
		PI 18:0/18:0	1.36	0.015
		PI 16:0/18:0*	1.40	0.044
	Lysophosphatidylcholine (LPC)	LPC 20:0	0.76	0.018
	Lysophosphatidylethanolamine (LPE)	LPE 18:2	0.56	0.048
Sphingolipis (SP)	Ceramides (Cer)	Cer d18:1/18:1	1.26	0.006
		Cer d18:1/16:0	1.37	0.017
	Hexosylceramides (HexCer)	HexCer d18:1/16:1	1.28	0.013
		HexCer d18:1/14:0	1.38	0.011
		HexCer d18:1/18:2	1.80	0.013
Sterol lipids (ST)	Cholesteryl ester (Chol. Esters)	CE 18:2	0.73	0.002
