# Supplementary material for: Targeted Lipidomics and Inflammation Response to Six Weeks of Sprint Interval Training in Male Adolescents
Source: Int J Environ Res Public Health. 2023 Feb 14;20(4):3329. doi: 10.3390/ijerph20043329 (PMC9963480; doi:10.3390/ijerph20043329)
Supplement: Supplementary file 1 [file ijerph-20-03329-s001.zip › Supplementary Table S2.pdf]

Table S2. Spearman correlations between changes in differential serum lipids and inflammatory markers

Inflammatory Markers	Lipid Name	Correlation	*p*-Value
CRP ↓	HexCer d18:1/16:1 ↑	0.94	0.000
	LPC 20:0 ↓	0.73	0.007
	HexCer d18:1/18:2 ↑	0.64	0.024
IL-1β ↓	HexCer d18:1/16:1 ↑	0.69	0.014
	LPC 20:0 ↓	0.60	0.038
IL-2 ↓	Docosahexaenoic acid(C22:6)	0.66	0.018
IL-4 ↓	HexCer d18:1/16:1 ↑	0.97	0.000
	HexCer d18:1/18:2 ↑	0.82	0.001
	LPC 20:0 ↓	0.66	0.018
	Eicosapentaenoic acid(C20:5) ↓	0.65	0.022
IL-6 ↑	LPE 18:2 ↓	-0.74	0.006
	Cer d18:1/16:0 ↑	0.71	0.010
	DG 18:0_18:0 ↑	0.69	0.014
	Octadecatetraenoic acid(C18:4) ↓	0.64	0.026
	MG 22:5 ↑	0.59	0.045
IL-10 ↓	HexCer d18:1/16:1 ↑	0.66	0.020
	Docosahexaenoic acid(C22:6) ↓	0.60	0.039
TGF-β ↓	HexCer d18:1/16:1 ↑	0.78	0.003
	LPC 20:0 ↓	0.61	0.036
TNF-α ↓	PI 18:0/18:0 ↑	0.59	0.045
IL-10/TNF-α ↑	Docosahexaenoic acid(C22:6) ↓	0.69	0.013
